# ﻿Misleading fruits: The non-monophyly of *Pseudopiptadenia* and *Pityrocarpa* supports generic re-circumscriptions and a new genus within mimosoid legumes

**DOI:** 10.3897/phytokeys.205.82275

**Published:** 2022-08-22

**Authors:** Leonardo M. Borges, Peter W. Inglis, Marcelo F. Simon, Pétala Gomes Ribeiro, Luciano P. de Queiroz

**Affiliations:** 1 Universidade Federal de São Carlos, Departamento de Botânica, Rodovia Washington Luís, Km 235, São Carlos, SP, 13565-905, Brazil Universidade Federal de São Carlos São Carlos Brazil; 2 Embrapa Recursos Genéticos e Biotecnologia, Parque Estação Biológica, Caixa Postal 02372, Brasília, DF, 70770-917, Brazil Embrapa Recursos Genéticos e Biotecnologia, Parque Estação Biológica Brasília Brazil; 3 Universidade Estadual de Feira de Santana, Departamento de Ciências Biológicas. Av. Transnordestina s.n., Novo Horizonte, Feira de Santana, BA, 44036-900, Brazil Universidade Estadual de Feira de Santana Feira de Santana Brazil

**Keywords:** Caesalpinioideae, Fabaceae, Leguminosae, *Parapiptadenia*, Stryphnodendron clade, tropical America

## Abstract

Generic delimitation in *Piptadenia* and allies (mimosoid legumes) has been in a state of flux, particularly caused by over-reliance on fruit and seed morphology to segregate species out of *Piptadenia* into the genera *Parapiptadenia*, *Pityrocarpa* and *Pseudopiptadenia*. Although supporting their segregation from *Piptadenia*, previous phylogenetic analyses suggested that some of these segregated genera are not monophyletic. Here, we test the monophyly of *Parapiptadenia*, *Pityrocarpa* and *Pseudopiptadenia* with dense taxon sampling across these genera, including the type species of each genus. Our analysis recovers *Parapitadenia* as monophyletic, but places *Pseudopiptadenia* species in two distinct lineages, one of which includes all three species of *Pityrocarpa*. Given that the type species of both *Pseudopiptadenia* and *Pityrocarpa* are nested in the same clade, we subsume *Pseudopiptadenia* under the older name *Pityrocarpa*. The remaining *Pseudopiptadenia* species are assigned to the new genus *Marlimorimia*. Alongside high molecular phylogenetic support, recognition of *Parapiptadenia*, *Pityrocarpa* and *Marlimorimia* as distinct genera is also supported by combinations of morphological traits, several of which were previously overlooked.

## ﻿Introduction

Generic delimitation in the mimosoid legumes is being continually revised, notably across the informal Piptadenia group sensu Lewis and Elias (1981), which included *Anadenanthera* Speg., *Microlobius* C. Presl, *Mimosa* L., *Parapiptadenia* Brenan, *Piptadenia* Benth., *Pityrocarpa* (Benth.) Britton & Rose, *Pseudopiptadenia* Rauschert and *Stryphnodendron* Mart. Most of the proposed generic re-circumscriptions within the Piptadenia group have involved segregating species out of *Piptadenia*, which was morphologically poorly-defined (Brenan 1955) and is known to be polyphyletic (Luckow et al. 2003; Jobson and Luckow 2007; Simon et al. 2016; Ribeiro et al. 2018). While previous phylogenetic and phylogenomic analyses confirm the segregation of *Parapiptadenia*, *Pityrocarpa* and *Pseudopiptadenia* and place them together with *Stryphnodendron* and *Microlobius* in the Stryphnodendron clade sensu Koenen et al. (2020), the monophyly of these three genera is still uncertain because of incomplete taxon sampling in previous analyses (Simon et al. 2016; Koenen et al. 2020; Ringelberg et al. 2022).

Species of *Parapiptadenia*, *Pityrocarpa* and *Pseudopiptadenia* are trees inhabiting Neotropical rain forests and seasonally dry tropical forests and woodlands (SDTFWs sensu Queiroz et al. 2017), with the majority of species in South America and just two taxa in North America (Pi.obliqua(Pers.)Brenanvar.obliqua and *Ps.psilostachya* (DC.) G.P. Lewis & M.P. Lima) (Brenan 1955, 1963; Rauschert 1982; Lima and Lima 1984; Lewis and Lima 1991; Queiroz 2009). Their bipinnate leaves vary widely in the number of pinnae, as well as leaflet number, size and shape. Flowers are pentamerous, dialipetalous or gamopetalous and arranged in elongated spikes. The diverse fruits and seeds have been the most prominent traits used to define each genus (Brenan 1955; Lewis and Elias 1981). *Parapiptadenia* includes six species with plano-compressed fruits opening along both sutures (typical legumes) and flat, compressed, narrowly-winged seeds lacking a pleurogram. Eleven species with similar seeds, but with follicles (fruits splitting along the upper suture only) were placed in *Pseudopiptadenia* (Rauschert 1982; Lewis and Lima 1991). The three species in *Pityrocarpa*, which was first proposed as a section of *Piptadenia* (Bentham 1842), differ from the other two genera by their regularly constricted moniliform legumes and lentiform whitish seeds with an U-shaped pleurogram (Jobson and Luckow 2007).

**Figure 1.**
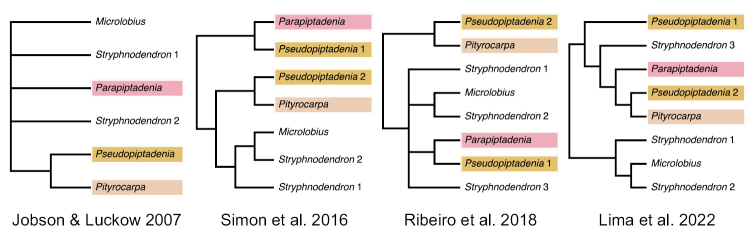
Topological differences amongst phylogenetic analyses of the Stryphnodendron clade.

The first phylogenetic analysis including these three genera recovered each as monophyletic, with *Pseudopiptadeniacontorta* (DC.) G.P. Lewis & M.P. Lima and *Ps.psilostachya* forming a clade sister to *Pityrocarpa* (three species sampled), while the relationship of *Parapiptadenia* (three species sampled) to other genera was uncertain (Fig. 1; Jobson and Luckow 2007). The relationships amongst these genera and the putative monophyly of *Pseudopiptadenia* were later questioned by analyses with larger DNA sequence datasets and increased taxon sampling (Simon et al. 2016; Ribeiro et al. 2018). In these analyses, *Parapiptadenia* (four species sampled) emerged as sister to a clade including all sampled species of *Pseudopiptadenia* (five species, including *Ps.contorta* and *Ps.psilostachya*), except *Ps.brenanii* G.P. Lewis & M.P. Lima, which was sister to *Pityrocarpa* (Fig. 1). This latter clade appeared more closely related to *Stryphnodendron* and *Microlobius* than to the group formed by *Parapiptadenia* and *Pseudopiptadenia.* Phylogenomic analyses with sparse taxonomic sampling recovered slightly different relationships between these three genera (Fig. 1), but reinforced the non-monophyly of *Pseudopiptadenia* (Lima et al. 2022; Ringelberg et al. 2022).

While it is clear that the non-monophyly of *Pseudopiptadenia* means that taxonomic adjustments are needed, the type species of the genus, *Ps.leptostachya*, has not been included in any previous phylogenetic analyses, raising doubts about its placement and, hence, about which generic name should be applied to the clade containing that species. In this study, we infer the phylogenetic relationships between *Parapiptadenia*, *Pityrocarpa* and *Pseudopiptadenia* using near-complete taxon sampling, including the type species of all three genera, and re-evaluate the circumscriptions of these genera, based on the resulting phylogenetic hypothesis.

## ﻿Materials and methods

### ﻿Phylogenetic inference

To further test the polyphyly of *Pseudopiptadenia* indicated by previous studies (Simon et al. 2016; Ribeiro et al. 2018; Ringelberg et al. 2022) and further investigate sister group relationships across the Stryphnodendron clade, we carried out phylogenetic analyses including near-complete sampling of species of *Parapiptadenia*, *Pityrocarpa*, *Pseudopiptadenia* and allies. Phylogenetic analyses were based on the nuclear ribosomal 5.8S subunit and internal transcribed spacer region (nrITS) and plastid regions *matK* and *trnD*-*trnT*. We generated 60 new sequences (21 nrITS, 23 *matK*, 16 *trnD*-*trnT*), including two accessions of *Ps.leptostachya*, the type species of *Pseudopiptadenia*, sampled here for the first time. Published sequences of other members of the Stryphnodendron clade and other genera were obtained from GenBank (Hughes et al. 2003; Simon et al. 2009; Simon et al. 2016; LPWG 2017; Ribeiro et al. 2018). Sampling comprised 60 accessions, including nine species (18 accessions) of *Pseudopiptadenia* (only the poorly known *Ps.colombiana* and *Ps.pittieri* were not sampled), all three species of *Pityrocarpa* (six accessions), all six known species of *Parapipitadenia* (11 accessions), plus representatives of the allied genera *Microlobius* (monospecific; two accessions) and *Stryphnodendron* (14 accessions, including members of the three major lineages of this non-monophyletic genus; see Lima et al. 2022). A selection of mimosoid lineages closely related to the Stryphnodendron clade (Jobson and Luckow 2007; Simon et al. 2016; Ribeiro et al. 2018; Ringelberg et al. 2022) were included as outgroups. Voucher details and GenBank accession numbers are provided in Table 1 and in the Suppl. material 1.

**Table 1.** Voucher information and GenBank accession numbers for taxa used in this study. Newly-generated sequences are in bold. See the Suppl. material 1 for a digital version.

Taxon	Voucher	Herbarium	nrITS	*matK*	*trnD*-*trnT*
*Ingaedulis* Mart.	*Queiroz 13797*; *Pennington 13282*	HUEFS; K	JX870764	AF523078	JQ417383
*Lachesiodendronviridiflorum* (Kunth) P.G. Ribeiro, L.P. Queiroz & Luckow	*Queiroz 13090*	HUEFS	MG001274	MG001286	MG001305
*Microlobiusfoetidus* (Jacq.) M. Sousa & G. Andrade	*Hughes 2150*	FHO	KT364047	KT364172	FJ981976
*Microlobiusfoetidus* (Jacq.) M. Sousa & G. Andrade	*Macqueen 432*	FHO	AF458783	AF523095	(No data)
*Mimosapalmeri* Rose	*Simon 823*	FHO	KT364059	KT364212	FJ982142
*Mimosapigra* L.	*Hughes 2414*	FHO	KT364060	KT364213	FJ982148
*Mimosaursina* Mart.	*Simon 704*	CEN	KT364061	KT364210	FJ982217
*Parapiptadeniablanchetii* (Benth.) Vaz & M.P. Lima	*Queiroz 15358*	HUEFS	**OM575100**	**ON409904**	**ON409927**
*Parapiptadeniablanchetii* (Benth.) Vaz & M.P. Lima	*Thomas 12372*	NY	**OM575099**	**ON409905**	(No data)
*Parapiptadeniaexcelsa* (Griseb.) Burkart	*Hughes 2425*	FHO	KT364062	KT364160	FJ982235
*Parapiptadeniailheusana* G.P. Lewis	*Neves 1659*	RB	**OM575101**	KY046081	**ON409928**
*Parapiptadeniapterosperma* (Benth.) Brenan	*Cardoso 2359*	HUEFS	**OM575102**	**ON409906**	**ON409929**
*Parapiptadeniapterosperma* (Benth.) Brenan	*Ribeiro 902*	HUEFS	MG001260	**ON409910**	MG001292
*Parapiptadeniarigida* (Benth.) Brenan	*Marestoni 26*	HUEFS	MG001261	**ON409909**	(No data)
*Parapiptadeniazehntneri* (Harms) M.P. Lima & H.C. Lima	*Cotarelli 2029*	HUEFS	**OM575104**	**ON409907**	(No data)
*Parapiptadeniazehntneri* (Harms) M.P. Lima & H.C. Lima	*Pereira-Silva 3102*	CEN	KT364063	KT364063	KT364108
*Parapiptadeniazehntneri* (Harms) M.P. Lima & H.C. Lima	*Queiroz 10974*	HUEFS	**OM575105**	**ON409908**	(No data)
*Parapiptadeniazehntneri* (Harms) M.P. Lima & H.C. Lima	*Queiroz 15692*	HUEFS	**OM575106**	KX302341	(No data)
*Piptadeniagonoacantha* (Mart.) J.F. Macbr.	*Simon 735*	FHO	KT364065	DQ790620	FJ982238
*Piptadeniastipulacea* (Benth.) Ducke	*Simon 702*; *Queiroz 3115*	CEN; HUEFS	KT386296	DQ790634	FJ982239
*Pityrocarpaleucoxylon* (Barneby & J.W. Grimes) Luckow & R.W. Jobson	*Fernandez 2909*	NY	(No data)	DQ790622	(No data)
*Pityrocarpamoniliformis* (Benth.) Luckow & R.W. Jobson	*Melo 7518*	HUEFS	(No data)	**ON409911**	**ON409936**
*Pityrocarpamoniliformis* (Benth.) Luckow & R.W. Jobson	*Queiroz 9084*	HUEFS	ON191501	**ON409912**	(No data)
*Pityrocarpamoniliformis* (Benth.) Luckow & R.W. Jobson	*Way 2449*	K	KT364067	KT364162	FJ982242
Pityrocarpaobliqua(Pers.)Brenansubsp.brasiliensis (G.P. Lewis) Luckow & R.W. Jobson	*Queiroz 12903*	HUEFS	**ON191500**	**ON409920**	(No data)
Pityrocarpaobliqua(Pers.)Brenansubsp.obliqua	*Macqueen 439*	FHO	KT364068	KT364206	FJ982243
*Pseudopiptadeniabahiana* G.P. Lewis & M.P. Lima	*Melo 138*	HUEFS	**OM575115**	**ON409916**	**ON409930**
*Pseudopiptadeniabahiana* G.P. Lewis & M.P. Lima	*Queiroz 15381*	HUEFS	MG001277	MG001290	**ON409931**
*Pseudopiptadeniabahiana* G.P. Lewis & M.P. Lima	*Queiroz 15504*	HUEFS	**OM575114**	**ON409917**	**ON409932**
*Pseudopiptadeniabrenanii* G.P. Lewis & M.P. Lima	*Borges 680*	SPF	KT364069	(No data)	KT364111
*Pseudopiptadeniabrenanii* G.P. Lewis & M.P. Lima	*Cardoso 2807*	HUEFS	**OM575108**	**ON409914**	**ON409937**
*Pseudopiptadeniabrenanii* G.P. Lewis & M.P. Lima	*Harley 56005*	HUEFS	**OM575109**	**ON409915**	(No data)
*Pseudopiptadeniabrenanii* G.P. Lewis & M.P. Lima	*Queiroz 15585*	HUEFS	MG001278	**ON409913**	**ON409938**
*Pseudopiptadeniacontorta* (DC.) G.P. Lewis & M.P. Lima	*Queiroz 15507*	HUEFS	(No data)	KT364155	KT364113
*Pseudopiptadeniacontorta* (DC.) G.P. Lewis & M.P. Lima	*Queiroz 15582*	HUEFS	MG001279	KX302348	MG001308
*Pseudopiptadeniainaequalis* (Benth.) Rauschert	*Lima 7790*	RB	**OM575111**	**ON409921**	**ON409939**
*Pseudopiptadenialeptostachya* (Benth.) Rauschert	*Lima 8231*	RB	**OM575113**	**ON409922**	**ON409940**
*Pseudopiptadenialeptostachya* (Benth.) Rauschert	*Lima 8326*	RB	**OM575112**	**ON409923**	**ON409941**
*Pseudopiptadeniapsilostachya* (DC.) G.P. Lewis & M.P. Lima	*Simon 1245*	CEN	KT364070	KT364170	KT364114
*Pseudopiptadeniaschumanniana* (Taub.) G.P. Lewis & M.P. Lima	*Lima 7938*	RB	**OM575110**	**ON409924**	**ON409942**
*Pseudopiptadenia* sp.	*Neves 1675*	RB	**OM575116**	**ON409918**	**ON409933**
*Pseudopiptadenia* sp.	*Ribeiro 351*	HUEFS	**OM575117**	**ON409919**	(No data)
*Pseudopiptadeniasuaveolens* (Miq.) J.W. Grimes = *P.psilostachya*	*Moacir & Clovis sn*	IAN	**OM575119**	**ON409925**	**ON409934**
*Pseudopiptadeniawarmingii* (Benth.) G.P. Lewis & M.P. Lima	*Queiroz 12761*	HUEFS	**OM575118**	**ON409926**	**ON409935**
*Senegaliamacrostachya* (Rchb. ex DC.) Kyal. & Boatwr.	*Miller 1322*	CANB	KY688790	KY688920	(No data)
*Senegalianigrescens* (Oliv.) P.J.H. Hurter	*Maurin 255*	JRAL	JQ265858	GQ872237	(No data)
*Stryphnodendronadstringens* (Mart.) Coville	*Souza 29702*	ESA	KT364072	KT364198	KT364116
*Stryphnodendroncoriaceum* Benth.	*Scalon 718*	ESA	(No data)	KT364200	KT364120
*Stryphnodendronduckeanum* Occhioni	*Simon 1343*	CEN	KT364076	(No data)	KT364122
*Stryphnodendronfissuratum* E.M.O. Martins	*Ivanauskas sn*	ESA	KT364077	KT364175	KT364124
*Stryphnodendronforeroi* E.M.O. Martins	*Assis 1143*	SPF	KT364079	KT364201	KT364126
*Stryphnodendrongracile* Rizzini & Heringer	*Scalon 458*	ESA	KT364080	KT364177	KT364127
*Stryphnodendronobovatum* Benth.	*Scalon 712*	ESA	KT364081	KT364182	KT364130
*Stryphnodendronocchionianum* E.M.O. Martins	*Simon 1597*	CEN	KT364083	(No data)	KT364132
*Stryphnodendronpaniculatum* Poepp.	*Simon 1058*	CEN	KT364084	(No data)	KT364133
*Stryphnodendronpolyphyllum* Mart.	*Mello-Silva 2659*	SPF	KT364086	KT364184	KT364136
*Stryphnodendronpulcherrimum* (Willd.) Hochr.	*Simon 980*	CEN	KT364087	(No data)	KT364137
*Stryphnodendronroseiflorum* (Ducke) Ducke	*Scalon 728*	ESA	KT364090	KT364193	KT364143
*Stryphnodendronrotundifolium* Mart.	*Scalon 715*	ESA	KT364094	KT364194	KT364147
*Stryphnodendronvelutinum* Scalon	*Scalon 719*	ESA	KT364101	KT364187	KT364153

Total DNA was extracted from about 20 mg of silica gel-dried leaf material using a modified CTAB-based protocol (Inglis et al. 2018a). We checked DNA quality and integrity using agarose gel electrophoresis and DNA quantity and purity estimated by Nanodrop spectrophotometry (Thermo Scientific). Laboratory procedures, primer sequences and amplification protocols followed Inglis et al. (2018b) for nrITS and Simon et al. (2016) for *matK* and *trnD*-*trnT*. PCR products were prepared for direct Sanger sequencing using ExoSAP (ThermoFisher) and both DNA strands were sequenced using the Big Dye v.3.1 kit (Applied Biosystems), using the amplification primers. We obtained further sequences included in the analysis from GenBank (Table 1).

We assembled contigs using Geneious Prime 2021 (https://www.geneious.com) and aligned matrices with MAFFT v.7 (Katoh and Standley 2013). Maximum Likelihood (ML) phylogenetic analysis was performed using IQ-TREE (Nguyen et al. 2015), using 1000 ultrafast bootstrap replicates to estimate branch support (Hoang et al. 2018) and models estimated with ModelFinder (Kalyaanamoorthy et al. 2017). Trees were drawn with FigTree (http://tree.bio.ed.ac.uk/software/figtree/) and rooted using *Lachesiodendronviridiflorum* (Kunth) P.G. Ribeiro, L.P. Queiroz & Luckow, following Ringelberg et al. (2022). Analyses of individual loci produced similar topologies, although the plastid trees were substantially less well-resolved compared to the nrITS phylogeny. In the absence of major incongruence between individual gene trees, we inferred phylogenetic relationships with a concatenated dataset (nrITS, *matK*, *trnD*-*trnT*) containing 3280 bp and 13% of missing data and used it as the basis for proposing taxonomic rearrangements.

## ﻿Data Resources

The data underpinning the analysis reported in this paper are deposited in GitHub at https://doi.org/10.5281/zenodo.6611789

## ﻿Results and discussion

**Figure 2.**
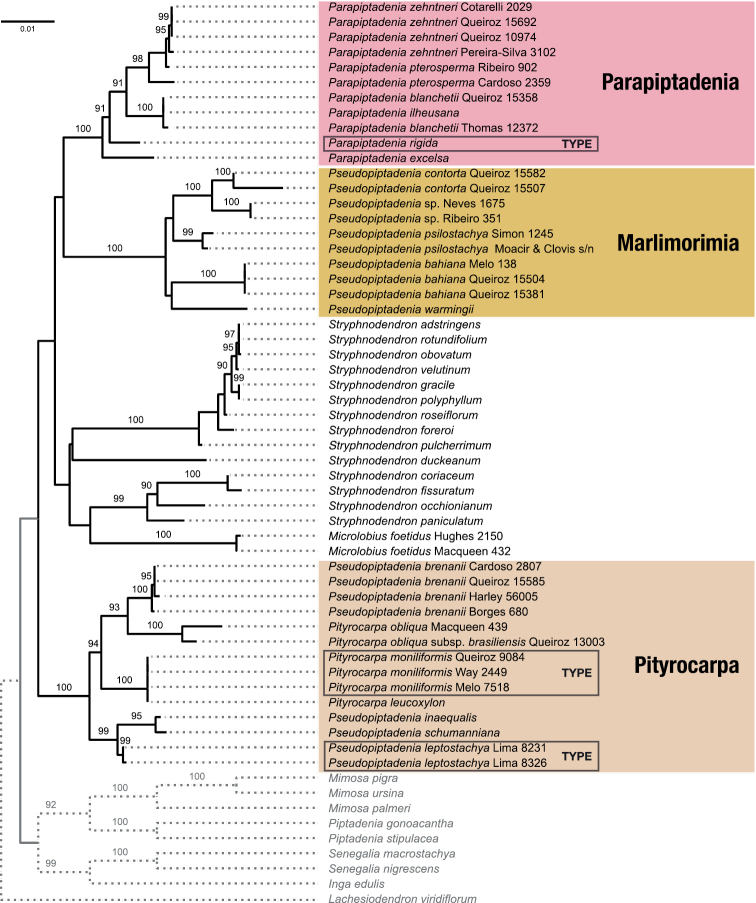
Phylogeny of the Stryphnodendron clade, based on Maximum Likelihood analysis of the concatenated nrITS, *matK* and *trnD*-*trnT* data. Significant ultrafast bootstrap values (> 90%) are given above branches. The tree was rooted using *Lachesiodendronviridiflorum*. Scale bar: expected number of changes per site; dotted branches not to scale.

Our densely sampled phylogenetic analysis recovers *Parapitadenia* as monophyletic, reinforces the non-monophyly of *Pseudopiptadenia* and shows that *Pityrocarpa* is also non-monophyletic (Fig. 2). Although the backbone of the phylogeny remains weakly-supported, the three main clades relevant to the delimitation of genera and the taxonomic decisions proposed here have full (100%) bootstrap support.

The first clade, hereafter referred to as *Pseudopiptadeniaproparte*, includes *Ps.bahiana* G.P. Lewis & M.P. Lima, *Ps.contorta*, *Ps.psilostachya*, *Ps.warmingii* (Benth.) G.P. Lewis & M.P. Lima and a putative new species yet to be described. The second clade, hereafter referred to as the *Pityrocarpa* clade, encompasses the remaining *Pseudopiptadenia* species, including the type species of the genus, *Ps.leptostachya*, intermixed with accessions of the three species of *Pityrocarpa*, including *Pi.moniliformis* (Benth.) Luckow & R.W. Jobson, the type species of *Pityrocarpa*.

The placement of *Parapiptadenia*, *Pityrocarpa*, and *Pseudopiptadenia* species in three distinct lineages and the robustly supported monophyly of *Parapiptadenia* agree with previous phylogenetic analyses (Fig. 1; Simon et al. 2016; Ribeiro et al. 2018; Lima et al. 2022; Ringelberg et al. 2022). However, the relationships amongst these three clades and other members of the Stryphnodendron clade remain unclear, because of the lack of support across the backbone of the clade (Figs 1 and 2) and disagreement with previous analyses. For example, although analyses of nuclear and plastid data (Simon et al. 2016; Ribeiro et al. 2018) also placed *Pseudopiptadenia p.p.* and *Parapiptadenia* in the same clade, this group could be sister to the remainder of the Stryphnodendron clade (Simon et al. 2016) or sister to the clade comprising *Stryphnodendron* and *Microlobius* (Ribeiro et al. 2018). Phylogenomic analyses based on 997 nuclear genes (Lima et al. 2022; Ringelberg et al. 2022) placed *Pseudopiptadenia p.p.* as sister to a group including *Stryphnodendronduckeanum* Occhioni f. plus a clade formed by *Parapiptadenia* and the *Pityrocarpa* clade. Furthermore, these nodes across the backbone of the Stryphnodendron clade show high gene tree conflict (Ringelberg et al. 2022) coinciding with very short branches and weak support in both conventional and phylogenomic analyses, highlighting the difficulties of inferring relationships across this part of the mimosoid phylogeny.

**Figure 3.**
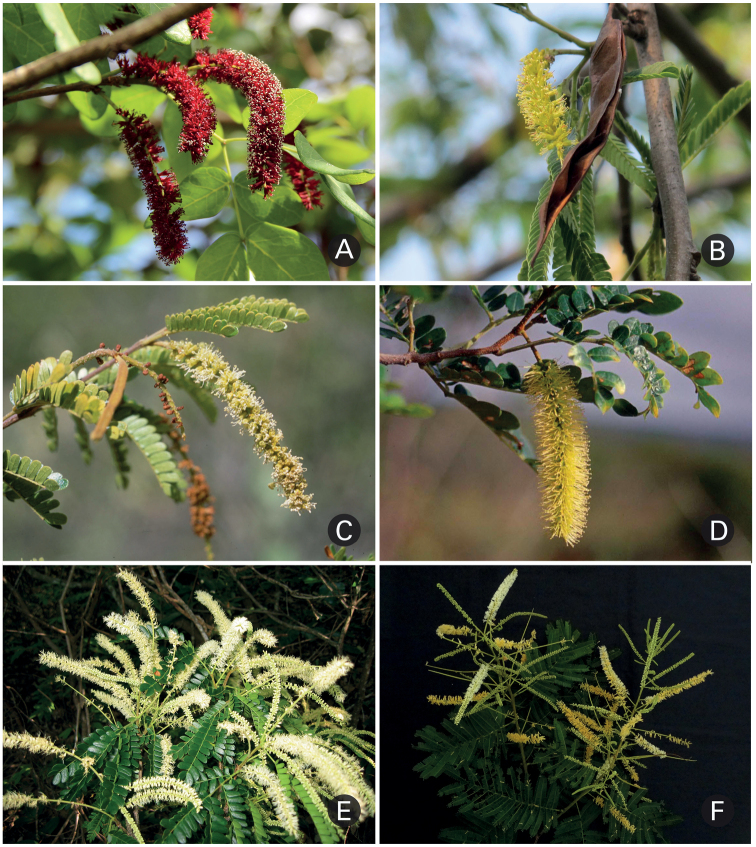
Inflorescences of *Parapiptadenia*, *Pityrocarpa* and *Marlimorimia***A***Parapiptadeniapterosperma* (Benth.) Brenan showing reddish inflorescences in the axils of coeval leaves **B***Pa.rigida* (Benth.) Brenan showing yellowish inflorescences and fruits with valves plicate above the seeds **C***Pityrocarpabrenanii* showing whitish, solitary spikes in the axils of coeval leaves **D***Pi.moniliformis* showing yellowish, solitary spikes in the axil of a coeval leaf **E***Marlimorimiabahiana* (G.P. Lewis & M.P. Lima) L.P. Queiroz & L.M. Borges, showing whitish spikes clustered in efoliate terminal pseudoracemes **F***Marlimorimia*sp. showing yellowish spikes clustered in efoliate terminal pseudoracemes (Photos: **A** PG Ribeiro; **B** RT de Queiroz **C–E** LP Queiroz; **F** G Siqueira).

Despite uncertainties regarding generic relationships, our results provide an additional example of how over-reliance on particular traits, in this case fruits and seeds (Brenan 1955; 1963; Lewis and Elias 1981), may lead to unnatural taxonomies. Presence of follicles and of flat and winged seeds, which were used to diagnose *Pseudopiptadenia*, are respectively shared by most lineages within the Stryphnodendron clade or homoplastic between *Pseudopiptadenia p.p.* and members of the *Pityrocarpa* clade. All this is not to say that fruits have no taxonomic significance, as the vast majority of *Parapiptadenia* species have distinctive legumes with valves plicate above the seeds, not seen in any other member of the Stryphnodendron clade. Nonetheless, most species in the *Pityrocarpa* clade, even though variable in seed morphology (flat and winged vs. lentiform and wingless), share a number of similarities, including the position of the extrafloral nectaries between or just below the first pair of pinnae; few pinnae pairs; inflorescence spikes in general solitary and axillary to coeval leaves; and bifoliolate seedlings (Fig. 3). These features are not shared with most *Pseudopiptadenia p.p.* species, which have extrafloral nectaries on the lower half of the petiole; many pairs of pinnae; inflorescence spikes arranged in complex efoliate synflorescences; and pinnate or bipinnate seedlings (see Table 2). Although fairly homogeneous within the *Pityrocarpa* clade and *Pseudopiptadenia p.p.*, the characters highlighted above sometimes vary amongst and within species, particularly in a context including *Parapiptadenia*. For example, solitary inflorescences occur in species of both *Parapiptadenia* and the *Pityrocarpa* clade, while *Pseudopiptadenia p.p.* species sometimes do not have spikes arranged in complex synflorescences (e.g. particular specimens of *Ps.bahiana* and *Ps.contorta*). Nonetheless, taken together, the traits highlighted here provide better recognition of these lineages as distinct genera than fruit morphology alone.

**Table 2.** Morphological comparison amongst *Parapiptadenia*, *Pityrocarpa* and *Marlimorimia*. Traits in bold highlight diagnostic features separating *Pityrocarpa* and *Marlimorimia*. EFN - Extrafloral nectary.

	*Parapiptadenia*	*Pityrocarpa*	*Marlimorimia*
**Pinnae number**	1–8	1–4 (rarely to 5 in *Pi.brenanii*)	5–10 or more (2–5 in *M.bahiana* and *M.colombiana*)
**Petiolar EFN position**	Variable across species	**Between or just below the first pair of pinnae**	**Between the base and the middle of the petiole**
**Spike arrangement**	Solitary, axillary or supra-axillary to coeval leaves	**Solitary (very rarely up to 2 in *Pi.moniliformis*), axillary to coeval leaves**	**2–many fasciculate and further arranged in efoliate terminal pseudoracemes or on efoliate nodes below mature leaves**
**Petals**	Reddish (yellowish in *Pa.excelsa* and *Pa.rigida*); united at the base (free in *Pa.rigida*)	**White to yellowish or greenish; free and glabrous (united in *Pi.leucoxylon*)**	**White to yellowish or greenish; united and pubescent**
**Fruit type (dehiscence)**	Legume	Follicle	Follicle
**Fruit shape**	Flat compressed, valves plicate above the seeds (except in Pa.excelsa)	Flat compressed, valves not plicate above the seeds	Flat compressed, valves not plicate above the seeds
**Fruit margins**	Straight to shallowly sinuous	**Deeply constricted (moniliform) (sinuous in *P.brenanii*).**	**Straight, (shallowly and irregularly sinuous in *M.bahiana*, *M.colombiana* and *M.warmingii*), sometimes constricted where seeds abort**
**Valve consistency and indumentum**	Thin, chartaceous, glabrous	**Thick, coriaceous, mostly pubescent**	**Thin, coriaceous (thicker and harder in *M.warmingii*), glabrous**
**Embryo plumule**	Developed and multifid	**Rudimentary (developed and multifid in *P.brenanii*)**	**Developed and multifid**
**Seedling eophylls**	Pinnate	**Bifoliolate**	**Pinnate or bipinnate**

These results from phylogenetic and morphological analyses provide robust support for re-circumscription of *Pseudopiptadenia* as it was traditionally conceived and also *Pityrocarpa*. Given that the type species of these two genera are nested in the same clade and that no morphological traits support the recognition of a narrow circumscription of *Pseudopiptadenia*, we subsume the name *Pseudopiptadenia* under *Pityrocarpa*, the oldest validly published generic name (Britton and Rose 1928; Lewis and Lima 1991; Turland et al. 2018). We assign the remaining *Pseudopiptadenia* species to the new genus *Marlimorimia*.

### ﻿Key to the genera Parapiptadenia, Pityrocarpa, and Marlimorimia. See Lima et al. (2022) for a key to all genera of the Stryphnodendron clade

**Table:** 

1	Petals reddish (yellowish in *Pa.excelsa* and *Pa.rigida*); fruit a legume (dehiscing along both sutures), the valves plicate above the seeds (except in *Pa.excelsa*)	***Parapiptadenia***
–	Petals white to yellowish or greenish; fruit a follicle (splitting along one suture only), the valves not plicate above the seeds	**2**
2	Petiolar nectary just below or between the first pair of pinnae; spikes solitary in axils of coevally developing leaves; petals free (united in *Pi.leucoxylon*) and glabrous; fruit margins deeply constricted (sinuous in *Pi.brenanii*)	***Pityrocarpa***
–	Petiolar nectary between the base and the middle of the petiole; spikes 2-many-fasciculate, the fascicles usually arranged in efoliate terminal pseudoracemes or on efoliate nodes below the leaves; petals united and pubescent; fruit margins straight or shallowly and irregularly sinuous, sometimes constricted where seeds abort	***Marlimorimia***

### ﻿Taxonomy

#### ﻿1. Pityrocarpa

Taxon classificationPlantaeFabalesFabaceae

(Benth.) Britton & Rose, N. Amer. Fl. 23(3): 190. 1928.

DE490BFF-A419-5D83-9D70-46B7A724DD13

Monoschisma Brenan, *Kew Bull.* 10(2): 179. 1955, *nom. inval.*, non Monoschisma Duby, Mém. Soc. Phys. Genève 19: 294. 1868. Type. Monoschismaleptostachyum (Benth.) Brenan, syn. nov.
Pseudopiptadenia Rauschert, Taxon 31(3): 559. 1982. Type. Pseudopiptadenialeptostachya (Benth.) Rauschert, syn. nov.

##### Basionym.

Piptadeniasect.Pityrocarpa Benth., J. Bot. (Hooker) 4: 339. 1842.

##### Type.

*Pityrocarpamoniliformis* (Benth.) Luckow & R.W. Jobson [≡ *Piptadeniamoniliformis* Benth., designated by Britton and Rose 1928].

##### Description.

Unarmed trees or shrubs. *Leaves* bipinnate; petiole with an extrafloral nectary between or shortly below the first pair of pinnae; pinnae 1–4 (5) pairs, exceptionally to 10 pairs in *Pi.leptostachya*; leaflets 1–10 pairs per pinna, rarely to 20 pairs (*Pi.brenanii* and *Pi.leptostachya*), mostly rhomboid sometimes also asymmetrically elliptical or lanceolate. *Inflorescences* spikes, solitary in the axils of coeval leaves, commonly pendulous. *Flowers* pentamerous; petals free (except possibly *Pi.leucoxylon*), glabrous; stamens 10, anther gland present; ovary shortly stipitate and included within or exserted from the corolla. *Fruit* a follicle, dehiscing along the lower suture, flat compressed, mostly moniliform, the margins deeply and regularly constricted, rarely sinuous margins and shallowly constricted (*Pi.brenanii* and occasionally in *Pi.leucoxylon*); valves stiffly coriaceous. *Seeds* mostly flat compressed with a coriaceous testa and a narrow marginal wing, lacking a pleurogram or, less frequently, ovoid or discoid with a hard, whitish testa, wingless and with a ‘U’-shaped pleurogram (*Pi.leucoxylon*, *Pi.moniliformis* and *Pi.obliqua*); embryo with a rudimentary plumule (except *Pi.brenanii*). *Seedlings* with bifoliolate eophylls.

##### Distribution.

*Pityrocarpa* is distributed in tropical America, from Mexico to southern Brazil and Paraguay. Most species occur in the Brazilian Atlantic rainforests (*Pi.inaequalis*, *Pi.leptostachya*, *Pi.schumanniana*), in the northern Amazonian rainforests (*Pi.leucoxylon*), in seasonally dry tropical forests and woodlands in the north-eastern Brazilian Caatinga (*Pi.brenanii*, *Pi.moniliformis*, Pi.obliquasubsp.brasiliensis), western Mexico (Pi.obliquasubsp.obliqua) or in Venezuelan savannas and Paraguayan Chaco (*Pi.moniliformis*).

##### Notes.

**Figure 4.**
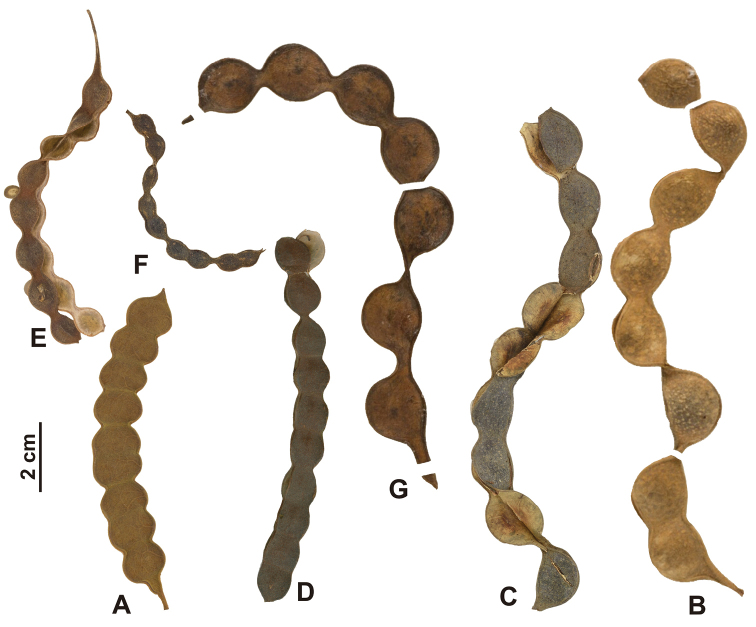
Fruits of *Pityrocarpa* species **A***Pi.brenanii* (from *Lewis et al. 1899*, NY) **B***Pi.inaequalis* (from *Moreira et al. 3*, F) **C***Pi.leptostachya* (from *Baez et al. 1174*, NY) **D***Pi.leucoxylon* (from *de Bruijn 1750*, NY) **E***Pi.moniliformis* (from *Nunes 597*, HUEFS) **F**Pi.obliquasubsp.brasiliensis (from *Mori 11837*, NY) **G***Pi.schumanniana* (from *Lima 2994*, RB).

As circumscribed here, *Pityrocarpa* includes seven species, all with a moniliform fruit, with the margins deeply constricted between the seeds (Fig. 4). This trait is shared by species formerly included in *Pityrocarpa* (sensu Jobson and Luckow 2007) and some species previously placed in the genus *Pseudopiptadenia* (sensu Lewis and Lima 1991). These two genera had been separated based on seed morphology, *Pityrocarpa* characterised by ovoid or discoid seeds with a hard, whitish seed coat and a ‘U’-shaped pleurogram, while *Pseudopiptadenia* included species with flat compressed and narrowly winged seeds with a coriaceous testa lacking a pleurogram. *Pityrocarpabrenanii* and *Pi.leucoxylon* have fruits with only shallowly sinuous margins, more similar to species of the genus *Marlimorimia*.

Besides sharing these fruit traits, *Pityrocarpa* species also have leaves with few pinnae (1 to 4 [5] pairs, rarely up to 10 pairs in *Pi.leptostachya*) and relatively large rhomboid leaflets compared to species of *Marlimorimia*. One exception are the leaves of *Pi.brenanii*, which are similar to those of *M.bahiana*. All species of *Pityrocarpa* present an extrafloral nectary between or shortly below the first pair of pinnae, in contrast to species of *Marlimorimia* that have the nectary below mid-petiole, frequently close to the pulvinus.

Floral traits, although previously disregarded as being generically diagnostic in the group, provide further evidence for the distinction between *Pityrocarpa* and *Marlimorimia*. The solitary inflorescence spikes in the axils of coevally developing leaves in *Pityrocarpa* contrast with the more complex synflorescences of *Marlimorimia* (Fig. 3; see notes under *Marlimorimia*). All species of *Pityrocarpa* have free and glabrous petals, except for *Pi.leucoxylon*, in which the petals are connate for a little over 1 mm (Barneby and Grimes 1984).

Lima (1985) and Lewis and Lima (1991) provided additional information on embryos and seedlings that are potentially useful for distinguishing *Pityrocarpa* from *Marlimorimia*. Embryos of *Pityrocarpa* species have a rudimentary plumule, while in *Marlimorimia*, the plumule is developed and multifid. This seems to be correlated with seedling morphology as the studied species of *Pityrocarpa* have bifoliolate eophylls and those of *Marlimorimia* species have pinnate or bipinnate eophylls (Lewis and Lima 1991). *Pityrocarpabrenanii*, however, has embryo morphology more similar to that reported for species of *Marlimorimia* (Lewis and Lima 1991).

#### ﻿1.1. Pityrocarpa brenanii

Taxon classificationPlantaeFabalesFabaceae

(G.P. Lewis & M.P. Lima) L.P. Queiroz & L.M. Borges comb. nov.

858B59B3-3691-5322-B6A2-868648A390D1

urn:lsid:ipni.org:names:77303780-1

##### Basionym.

*Pseudopiptadeniabrenanii* G.P. Lewis & M.P. Lima, Arch. Jard. Bot. Rio de Janeiro 30: 50–51. 1991.

##### Type.

Brasil, Bahia, *Harley et al. 21346* (holotype CEPEC; isotypes BR, K, M, MBM, MEXU, NY, RB, US).

#### ﻿1.2. Pityrocarpa inaequalis

Taxon classificationPlantaeFabalesFabaceae

(Benth.) L.P. Queiroz & Marc.F. Simon comb. nov.

6F53F79C-7E6E-5808-B6E3-5438F8B3A022

urn:lsid:ipni.org:names:77303781-1

Monoschisma inaequale (Benth.) Brenan, Kew Bull. 10(2): 179. 1955.
Pseudopiptadenia inaequalis (Benth.) Rauschert, Taxon 31(3): 559. 1982.

##### Basionym.

*Piptadeniainaequalis* Benth., J. Bot. (Hooker) 4: 339. 1842.

##### Type.

Brazil, Rio de Janeiro, *Pohl 1386* (lectotype K 000504704, designated here; isolectotype K 000504706).

#### ﻿1.3. Pityrocarpa leptostachya

Taxon classificationPlantaeFabalesFabaceae

(Benth.) L.P. Queiroz & P.G. Ribeiro comb. nov.

8A288014-0A12-598F-9636-964234200F62

urn:lsid:ipni.org:names:77303782-1

Monoschisma leptostachyum (Benth.) Brenan, Kew Bull. 10(2): 179. 1955.
Pseudopiptadenia leptostachya (Benth.) Rauschert, Taxon 31(3): 559. 1982.

##### Basionym.

*Piptadenialeptostachya* Benth., J. Bot. (Hooker) 4: 339. 1842.

##### Type.

Brasil, *Sellow s.n.* (Lectotype K 000504709, designated here; isolectoypes F 0360957F [fragment], K 000504710, TUB 009699).

##### Note.

Lewis and Lima (1991) unintentionally lectotypified this name by indicating the holotype to be at B and the isotype to be at K. However, the B specimen was destroyed and, hence, cannot serve as a lectotype. Moreover, K holds two duplicates of an un-numbered Sellow collection. Here, we chose the one previously belonging to Bentham’s herbarium as the lectotype.

#### ﻿1.4. Pityrocarpa leucoxylon

Taxon classificationPlantaeFabalesFabaceae

(Barneby & J.W. Grimes) Luckow & R.W. Jobson, Syst. Bot. 32(3): 573. 2007.

99EA921A-AF79-58D7-9C84-3DCCEC563FF6

##### Basionym.

*Piptadenialeucoxylon* Barneby & J.W. Grimes, Brittonia 36(3): 236–238. 1984.

##### Type.

Venezuela: Bolivar, *de Bruijn 1750* (holotype NY; isotypes MO, VEN, US).

#### ﻿1.5. Pityrocarpa moniliformis

Taxon classificationPlantaeFabalesFabaceae

(Benth.) Luckow & R.W. Jobson, Syst. Bot. 32(3): 573. 2007.

22F41D13-BBEF-51BA-8579-9639629C420C

Stryphnodendron piptadenioides E.M.O. Martins, Leandra 5(6): 90. 1975. Type. Brazil. Pernambuco, “lectum in silva pluviali ad S. José Belmonte”, Mata da Mina, 29 Oct 1971, *Ramalho 52* (holotype RFA 17173) .
Stryphnodendron consimile E.M.O. Martins, Leandra 5(6): 92. 1975. Type. Brazil. Piauí, “habitat in caatinga ad Paulistana”, Fazenda Altamira, 04 Nov 1974, *Lima 1330* (holotype RFA 17172).

##### Basionym.

*Piptadeniamoniliformis* Benth., *J. Bot.* (Hooker) 4: 339. 1842.

##### Type.

Brazil, Bahia, Serra de Jacobina, *Blanchet 2701* (lectotype K 000090193, designated here; isolectotypes F, K 000205897, MO, NY 00003233).

#### ﻿1.6. Pityrocarpa obliqua

Taxon classificationPlantaeFabalesFabaceae

(Pers.) Brenan, Kew Bull. 10(2): 176. 1955.

55050EA9-3933-5DF4-B6AB-794B0D4B1C72

Acacia thibaudiana DC., Prodr. 2: 456. 1825.
Piptadenia obliqua (Pers.) J.F. Macbr., Contr. Gray Herb. 59: 17. 1919.

##### Basionym.

*Sophoraobliqua* Pers., Syn. P*l.* 1: 452. 1805.

##### Type.

“Amer. australi?”, Herb. D. Thibaud. (not located).

**1.6.1. Pityrocarpaobliquasubsp.obliqua**

#### ﻿1.6.2. Pityrocarpa obliqua subsp. brasiliensis

Taxon classificationPlantaeFabalesFabaceae

(G.P. Lewis) Luckow & R.W. Jobson, Syst. Bot. 32(3): 573. 2007.

3E602A15-FCE0-56E0-9C6E-8DF5BE2F09BD

##### Basionym.

Piptadeniaobliquasubsp.brasiliensis G.P. Lewis, *Kew Bull.* 46(1): 160–162. 1991.

##### Type.

Brazil, Bahia, *Mori et al. 9519* (holotype CEPEC; isotypes HUEFS, K, NY).

#### ﻿1.7. Pityrocarpa schumanniana

Taxon classificationPlantaeFabalesFabaceae

(Taub.) L.P. Queiroz & L.M. Borges comb. nov.

3F6820DC-420D-5D50-96F7-61DA64A4D69E

urn:lsid:ipni.org:names:77303784-1

Pseudopiptadenia schumanniana (Taub.) G.P. Lewis & M.P. Lima, Arch. Jard. Bot. Rio de Janeiro 30: 53. 1991.

##### Basionym.

*Piptadeniaschumanniana* Taub., *Flora* 75: 75. 1892.

##### Type.

Brazil, “Brasilia austro-orientale”, Rio de Janeiro, *Glaziou 13774* (lectotype R 00008369, designated here; isolectotypes A 00064056, F 0058675F, K 000504703, MPU 016109, NY 00003244, NY 00003245, US 00001018, US 00997081).

#### ﻿2. Marlimorimia

Taxon classificationPlantaeFabalesFabaceae

L.P. Queiroz, L.M. Borges, Marc.F. Simon & P.G. Ribeiro gen. nov.

61737C47-E7E0-54E4-B3BA-221557AFC1F8

urn:lsid:ipni.org:names:77303785-1

Newtonia sect. Neonewtonia Burkart, Fl. Il. Catarin. fasc. LEGU: 285. 1979, syn. nov. Type. Newtonianitida (Benth.) Brenan (= Marlimorimiacontorta (DC.) L.P. Queiroz & P.G. Ribeiro).

##### Diagnosis.

*Marlimorimia* shares with *Pityrocarpa* the follicle, a fruit dehiscing along the lower suture only, and flat, compressed winged seeds, which lack a pleurogram. It can be differentiated from *Pityrocarpa* by the position of the extrafloral nectary on the petiole (from the base to the mid-petiole in *Marlimorimia* vs. between or just below the first pair of pinnae in *Pityrocarpa*); inflorescence spikes clustered in terminal pseudoracemes or in fascicles at efoliate nodes, surpassed by mature leaves (vs. solitary spikes in the axils of coeval leaves); petals united and joined into a gamopetalous corolla (vs. petals free and glabrous); and fruits with margins straight to shallowly sinuous (vs. margins deeply constricted).

##### Type.

*Marlimorimiacontorta* (DC.) L.P. Queiroz & P.G. Ribeiro

##### Description.

Unarmed trees. *Leaves* bipinnate; petiole with an extrafloral nectary well below the first pair of pinnae, close to the pulvinus, always below mid-petiole; pinnae 5–10 to many pairs per leaf (2–3 pairs in *M.colombiana* and 3–5 in *M.bahiana*); leaflets mostly > 10 pairs per pinna, (6–8 in *M.colombiana*), mostly oblong to linear from an asymmetrical base, rarely rhomboid (*M.bahiana*). *Inflorescences* spikes, grouped in fascicles, these being arranged in terminal pseudoracemes or forming clusters below the coeval leaves. *Flowers* pentamerous; petals united into a gamopetalous corolla, pubescent; stamens 10, anther gland present; ovary shortly stipitate and included or exserted from the corolla. *Fruit* a follicle, dehiscing along the lower suture, flat compressed, straight, curved or longitudinally twisted, the margins usually straight, rarely irregularly sinuous and only becoming constricted where the seeds fail to develop (*M.bahiana* and *M.warmingii*), valves coriaceous, thin or thick. *Seeds* flat compressed with a coriaceous testa, presenting a narrow or somewhat wider marginal wing, pleurogram lacking; embryo with a developed, multifid plumule (unknown in *M.colombiana* and *M.pittieri*). *Seedlings* with pinnate or bipinnate eophylls (unknown in *M.bahiana*, *M.colombiana* and *M.pittieri*).

##### Distribution.

*Marlimorimia* comprises six species with a bicentric distribution in the two main areas of tropical humid forests in South America. Three species occur in eastern Brazil, two of which are restricted to the Atlantic wet forests (*Marlimorimabahiana* and *M.warmingii*) and *M.contorta*, which extends to inland semi-deciduous forests. The three other species are distributed in northern South America. *Marlimorimiapsilostachya* is widely distributed across Amazonia, sparsely extending to Central America (Costa Rica) and *M.colombiana* and *M.pittieri* have restricted ranges in Colombia and Venezuela, respectively.

##### Etymology.

The genus *Marlimorimia* is named in honour of Dr. Marli Pires Morim, taxonomist at the Rio de Janeiro Botanical Garden, for her outstanding contribution to our knowledge of the diversity and taxonomy of Brazilian mimosoid legumes.

##### Notes.

The new genus *Marlimorimia* is proposed to accommodate a monophyletic group of species, previously classified in *Pseudopiptadenia* (sensu Lewis and Lima 1991; Luckow 2005), but which could not retain the genus name, because its type species is now included in *Pityrocarpa*.

Besides the molecular phylogenetic evidence, morphology also supports recognition of *Marlimorimia* as distinct from *Pityrocarpa*. *Marlimorimia* brings together most of the species formerly placed in *Pseudopiptadenia* which have multipinnate leaves, small oblong to linear leaflets and fruits with straight (or shallowly sinuous) margins. *Marlimorimiabahiana* and *M.colombiana*, however, have leaves with few pinnae and rhomboid leaflets.

Species of *Marlimorimia* have more complex inflorescences than those of *Pityrocarpa*. While the spikes of *Pityrocarpa* are solitary in the axils of coevally developing leaves, *Marlimorimia* species have spikes in fascicles of 2–3, which are arranged in terminal efoliate pseudoracemes or clustered on nodes below mature leaves (Fig. 3). Sometimes, as leaves expand, *Marlimorimia* synflorescences may resemble those of *Pityrocarpa* and *Parapiptadenia* (e.g. particular specimens of *M.contorta* such as *Hatschbach 50149* [NY]). Nonetheless, flowers of *Marlimorimia* have pubescent petals united into a gamopetalous corolla (vs. free glabrous petals in the majority of *Pityrocarpa* species).

**Figure 5.**
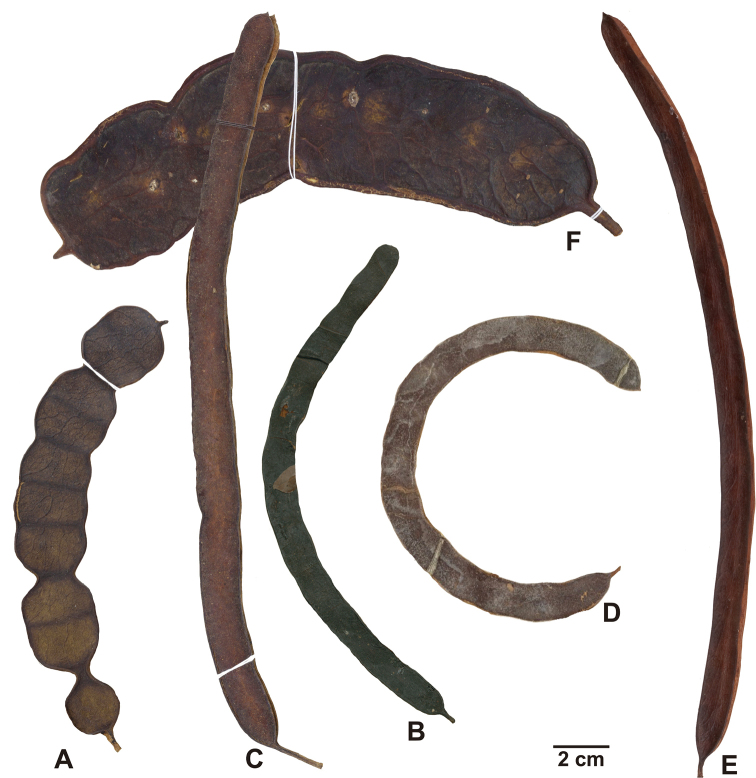
Fruits of *Marlimorimia* species **A***Marlimorimiabahiana* (from *Amorim 1009*, NY) **B***Marlimorimiacolombiana* (from *Killip 16268*, NY) **C***Marlimorimiacontorta* (from *Gomes 257*, NY) **D***Marlimorimiapittieri* (from *Guevara 1264*, F) **E***Marlimorimiapsilostachya* (from *Rabelo 2753*, NY) **F***Marlimorimiawarmingii* (from *Nunes et al. 2*, NY).

Two types of fruits are found in *Marlimorimia* (Fig. 5). Some species have long linear fruits, frequently curved or longitudinally twisted with straight margins (*M.colombiana*, *M.contorta*, *M.pittieri* and *M.psilostachya*), while *M.bahiana* and *M.warmingii* have oblong fruits with shallowly sinuous margins. The valves of the fruits are woody, although usually thin, becoming thicker and harder in *M.warmingii*.

The seeds of *Marlimorimia*, although superficially similar to those of most species of *Pityrocarpa*, have embryos with multifid plumules that result in seedlings with pinnate or bipinnate eophylls (Lima 1985; Lewis and Lima 1991).

#### ﻿2.1. Marlimorimia bahiana

Taxon classificationPlantaeFabalesFabaceae

(G.P. Lewis & M.P. Lima) L.P. Queiroz & L.M. Borges comb. nov.

8ABDAF17-E53A-57A1-B9D8-0C725BC81F24

urn:lsid:ipni.org:names:77303786-1

##### Basionym.

*Pseudopiptadeniabahiana* G.P. Lewis & M.P. Lima, Arch. Jard. Bot. Rio de Janeiro 30: 54–55. 1991.

##### Type.

Brasil, Bahia, *Mori & King 12223* (holotype CEPEC; isotypes HUEFS, K, NY, RB).

#### ﻿2.2. Marlimorimia colombiana

Taxon classificationPlantaeFabalesFabaceae

(Britton & Killip) L.P. Queiroz & Marc.F. Simon comb. nov.

0B8F897E-13A8-5B85-8BE1-C3F0B8EE0E85

urn:lsid:ipni.org:names:77303787-1

Pseudopiptadenia colombiana (Britton & Killip) G.P. Lewis

##### Basionym.

*Stryphnodendroncolombianum* Britton & Killip, Ann. New York Acad. Sci. 35(3): 155. 1936.

##### Type.

Colombia, Santander, *Killip & Smith 16268* (holotype NY 00003356; isotypes A, GH, US).

##### Notes.

In the absence of phylogenetic evidence, the petiolar extrafloral nectaries located at mid-petiole and fruits with straight to shallowly sinuous margins support the transfer of *Pseudopiptadeniacolombiana* to *Marlimorimia*.

#### ﻿2.3. Marlimorimia contorta

Taxon classificationPlantaeFabalesFabaceae

(DC.) L.P. Queiroz & P.G. Ribeiro comb. nov.

54281372-8BA0-585F-AD75-769AFA34581F

urn:lsid:ipni.org:names:77303788-1

Piptadenia nitida Benth., J. Bot. (Hooker) 4: 336. 1842.
Piptadenia contorta (DC.) Benth., Trans. Linn. Soc. Lond. 30: 368. 1875.
Newtonia nitida (Benth.) Brenan, Kew. Bull. 10 (2): 182. 1955.
Newtonia contorta (DC.) Burkart, Fl. Il. Catarin. fasc. LEGU: 289. 1979.
Pseudopiptadenia contorta (DC.) G.P. Lewis & M.P. Lima, Arch. Jard. Bot. Rio de Janeiro 30: 57. 1991.

##### Basionym.

*Acaciacontorta* DC., Prodr. 2: 470. 1825.

##### Type.

Brasil, Rio de Janeiro, *Raddi s.n.* (lectotype FI, designated here).

#### ﻿2.4. Marlimorimia pittieri

Taxon classificationPlantaeFabalesFabaceae

(Harms) L.P. Queiroz & L.M. Borges comb. nov.

3DDCBBBD-D7DA-52ED-B95F-4AFEBE689F6E

urn:lsid:ipni.org:names:77303789-1

Piptadenia similis Britton & Killip, Ann. New York Acad. Sci. 35(3): 156. 1936. Holotype Colombia, Barranquilla, *Elias 263* (US).
Pseudopiptadenia pittieri (Harms) G.P. Lewis, Kew Bull. 46(1): 118. 1991.

##### Basionym.

*Piptadeniapittieri* Harms, Notizbl. Bot. Gart. Berlin-Dahlem 8(71): 51–52. 1921.

##### Type.

Venezuela, Carabobo, *Pittier 8859* (lectotype US 00001013, designated here; isolectotypes GH 00064052, NY 00003236).

##### Notes.

Although *Pseudopiptadeniapittieri* was not included in the phylogenetic analyses, the presence of extrafloral nectaries at the base of the petiole, spikes arranged in pseudoracemes and fruits with straight margins support its transfer to *Marlimorimia*.

#### ﻿2.5. Marlimorimia psilostachya

Taxon classificationPlantaeFabalesFabaceae

(DC.) L.P. Queiroz & Marc.F. Simon comb. nov.

0BC8F15B-7E3A-52FC-95E6-D5457BA68FAE

urn:lsid:ipni.org:names:77303790-1

Piptadenia psilostachya (DC.) Benth., J. Bot. (Hooker) 4: 336. 1842.
Piptadenia suaveolens Miq., Linnaea 18: 589–590. 1845. Type Surinam, Bergendaal, *Focke 936* (holotype U).
Newtonia psilostachya (DC.) Brenan, Kew. Bull. 10 (2): 182. 1955.
Newtonia suaveolens (Miq.) Brenan, Kew. Bull. 10 (2): 182. 1955.
Pseudopiptadenia psilostachya (DC.) G.P. Lewis & M.P. Lima, Arch. Jard. Bot. Rio de Janeiro 30: 55. 1991.
Pseudopiptadenia suaveolens (Miq.) J.W. Grimes, Brittonia 45(1): 27. 1993.

##### Basionym.

*Acaciapsilostachya* DC., Prodr. 2: 457. 1825.

##### Type.

French Guiana, Cayenne, *Martin 2* (lectotype K 000504699, designated by Lewis & Lima 1991; isolectotype P 02930999).

##### Notes.

Contrary to Grimes (1993), who recognised *Pseudopiptadeniapsilostachya* and *Ps.suaveolens* as distinct species, we agree with Lewis and Lima (1991) on the synonymisation of *Ps.suaveolens* under *M.psilostachya*. These plants grow sympatrically and the traits used by Grimes (1993) to support recognition of two species are too variable to be diagnostic.

#### ﻿2.6. Marlimorimia warmingii

Taxon classificationPlantaeFabalesFabaceae

(Benth.) L.P. Queiroz & P.G. Ribeiro comb. nov.

46875E4E-9CB2-501E-A5B5-FFE74C3B3993

urn:lsid:ipni.org:names:77303791-1

Piptadenia glaziovii Harms, Repert. Spec. Nov. Regni Veg. 17: 203. 1921. Type. Brasil, Rio de Janeiro, Serra da Estrela, *Glaziou 8440* (lectotype K, designated by Lewis and Lima 1991).
Newtonia glaziovii (Harms) Burkart ex Barth & Yoneshigue, Mem. Inst. Oswaldo Cruz 64: 102. 1966.
Newtonia warmingii (Benth.) G.P. Lewis, Legumes of Bahia p. 111. 1987.
Pseudopiptadenia warmingii (Benth.) G.P. Lewis & M.P. Lima, Arch. Jard. Bot. Rio de Janeiro 30: 54. 1991.

##### Basionym.

*Mimosawarmingii* Benth., Trans. Linn. Soc. London 30(3): 413. 1875.

##### Type.

Brasil, Minas Gerais, Lagoa Santa, *Warming s.n.* (lectotype K 000504702, designated by Lewis and Lima 1991).

## Supplementary Material

XML Treatment for Pityrocarpa

XML Treatment for Pityrocarpa brenanii

XML Treatment for Pityrocarpa inaequalis

XML Treatment for Pityrocarpa leptostachya

XML Treatment for Pityrocarpa leucoxylon

XML Treatment for Pityrocarpa moniliformis

XML Treatment for Pityrocarpa obliqua

XML Treatment for Pityrocarpa obliqua subsp. brasiliensis

XML Treatment for Pityrocarpa schumanniana

XML Treatment for Marlimorimia

XML Treatment for Marlimorimia bahiana

XML Treatment for Marlimorimia colombiana

XML Treatment for Marlimorimia contorta

XML Treatment for Marlimorimia pittieri

XML Treatment for Marlimorimia psilostachya

XML Treatment for Marlimorimia warmingii

## Supplementary materials

Supplementary material 1 Voucher information and GenBank accession numbers (internal transcribed spacer-ITS sequences) for taxa used in this study Data type excel file Explanation note Voucher information and GenBank accession numbers for taxa used in this study. https://binary.pensoft.net/file/732771This dataset is made available under the Open Database License (http://opendatacommons.org/licenses/odbl/1.0/). The Open Database License (ODbL) is a license agreement intended to allow users to freely share, modify, and use this Dataset while maintaining this same freedom for others, provided that the original source and author(s) are credited.Leonardo M. Borges, Peter W. Inglis, Marcelo F. Simon, Pétala Gomes Ribeiro, Luciano P. de Queiroz
